# Cross-sectional analysis of gut microbiome diversity with progression of Alzheimer's disease

**DOI:** 10.6026/973206300213329

**Published:** 2025-09-30

**Authors:** Prashant Sundeep Gupta, Vedanth Mayakrishna Padmakumar, Omar Imtiaz Usmani

**Affiliations:** 1 Department of Medicine, Surat Municipal Institute of Medical Education and Research, Smimer, Gujarat, India; 2 Department of Casualty, Casualty Medical Officer, Kerala, India; 3 Department of General Medicine, Era's Lucknow Medical college Lucknow, Uttar Pradesh, India

**Keywords:** Gut microbiome, Alzheimer's disease, cognitive decline, dysbiosis, 16S rRNA sequencing

## Abstract

The relationship between gut microbiome diversity and stages of Alzheimer's disease (AD) progression is of interest. Hence, a total of 124 participants, including cognitively normal controls and patients with mild, moderate, and severe AD, were assessed for microbiome composition using 16S rRNA sequencing. Results revealed significantly reduced microbial diversity and altered bacterial profiles, notably lower levels of *Bifidobacterium* and *Faecalibacterium* and increased *Proteobacteria*, in advanced AD stages. Correlations were observed between cognitive declines and reduced alpha diversity. Thus, we show gut dysbiosis may play a contributory role in Alzheimer's pathology.

## Background:

Over the past decades, accumulating evidence suggests that the gut microbiome exerts a key role in Alzheimer's disease (AD). The Alzheimer's Association Workgroup is updating the diagnostic criteria for AD, which changed the profiles and categorization of biomarkers from "AT (N)" to "ATNIVS" [1]. Alzheimer's disease (AD) is a progressive neurodegenerative disorder characterized by memory loss, cognitive decline and behavioral disturbances [2]. While its pathogenesis is multifactorial, emerging evidence highlights a potential link between the gut-brain axis and neurodegeneration [3]. The gut microbiome a dynamic ecosystem of microorganisms residing in the gastrointestinal tract-has been implicated in modulating immune responses, inflammation and neuronal signaling [4]. Recent studies suggest that gut dysbiosis, or an imbalance in microbial composition, may influence the onset and progression of Alzheimer's disease through various mechanisms, including increased intestinal permeability, systemic inflammation and altered production of neuroactive metabolites such as short-chain fatty acids (SCFAs) [5]. Specific bacterial taxa, including *Bifidobacterium*, Lactobacillus and *Faecalibacterium*, are believed to confer neuroprotective effects, while the overgrowth of pro-inflammatory bacteria like *Proteobacteria* may exacerbate cognitive decline [6]. Despite increasing interest, there remains a paucity of clinical data correlating distinct patterns of gut microbial diversity with the severity of cognitive impairment in Alzheimer's patients [7]. Amyloid beta oligomers and plaques, tau aggregates, and neuroinflammation play a critical role in neurodegeneration and impact clinical AD progression. The upstream modulators of these pathological features have not been fully clarified, but recent evidence indicates that the gut microbiome (GMB) may have an influence on these features and therefore may influence AD progression in human patients [8]. Therefore, it is of interest to analyze gut microbiome diversity across different stages of AD to better understand its potential role in disease progression and to identify microbial signatures associated with cognitive decline.

## Methods:

This cross-sectional study was conducted at a tertiary care neurology center over a period of eight months. A total of 124 participants aged 60 years and above were enrolled, comprising four groups: cognitively normal controls (n=31), mild Alzheimer's disease (n=33), moderate Alzheimer's disease (n=32), and severe Alzheimer's disease (n=28), based on MMSE (Mini-Mental State Examination) scores and clinical evaluation using NIA-AA criteria. Participants with gastrointestinal diseases, recent antibiotic or probiotic use (within 3 months), and other neurodegenerative conditions were excluded. Stool samples were collected from all participants under standardized conditions and stored at -80°C. Microbial DNA was extracted using the QIAamp DNA Stool Mini Kit. 16S rRNA gene sequencing targeting the V3-V4 regions was performed using Illumina MiSeq technology. Operational Taxonomic Units (OTUs) were clustered at 97% similarity using QIIME2, and taxonomic classification was performed using the Greengenes database. Microbial diversity was assessed via alpha diversity metrics (Shannon index, Chao1 richness) and beta diversity using Bray-Curtis dissimilarity. Taxonomic composition was analyzed at phylum and genus levels. Comparisons across groups were made using Kruskal-Wallis and ANOVA tests where appropriate. Spearman correlation was used to assess the relationship between MMSE scores and microbial diversity indices. A p-value of <0.05 was considered statistically significant.

## Results:

Analysis revealed a progressive decline in gut microbial diversity from cognitively normal individuals to those with severe Alzheimer's disease (AD). Alpha diversity indices (Shannon and Chao1) were significantly lower in moderate and severe AD groups. Additionally, taxonomic profiling showed increased abundance of pro-inflammatory bacteria (*Proteobacteria*, Escherichia) and reduced levels of beneficial genera (*Bifidobacterium*, *Faecalibacterium*) with advancing AD severity. Beta diversity plots showed distinct clustering of microbial communities by AD stage. Table 1 (see PDF) shows there was a clear decline in microbial alpha diversity across worsening stages of AD. Table 2 (see PDF) shows the relative abundance of Bacteroidetes and Firmicutes shifted significantly with disease progression. Table 3 (see PDF) shows the abundance of anti-inflammatory genera like *Faecalibacterium* showed a marked decrease. Table 4 (see PDF) shows beta diversity plots revealed distinct clustering by AD stage, indicating compositional differences. Table 5 (see PDF) shows lower MMSE scores were strongly correlated with reduced microbial diversity. Table 6 (see PDF) shows pro-inflammatory genera were more abundant in severe AD patients. Table 7 (see PDF) shows SCFA-producing bacteria showed a declining trend in advanced AD. Table 8 (see PDF) shows diversity was significantly lower in AD patients reporting constipation a common symptom in gut dysbiosis. Table 9 (see PDF) shows gender did not significantly impact microbial diversity in any group. Table 10 (see PDF) shows use of anti-dementia medications did not significantly alter microbiome diversity.

## Discussion:

This cross-sectional analysis reinforces the emerging hypothesis that gut microbiome alterations are closely associated with the progression of Alzheimer's disease (AD). Various treatment options for AD have been investigated to be involved in the gut microbiome [9]. A marked decline in alpha diversity and a shift in microbial composition were evident as cognitive function deteriorated. Beneficial bacteria such as *Faecalibacterium* and *Bifidobacterium*, known for their anti-inflammatory and neuroprotective properties, were significantly depleted in moderate and severe AD, suggesting a compromised gut-brain axis [10]. Simultaneously, the rise in *Proteobacteria* and Escherichia-taxa linked to pro-inflammatory states suggests that microbial imbalance may contribute to the neuroinflammatory processes underlying AD pathology [11]. The correlation between MMSE scores and microbial diversity indices underscores a potential role of gut microbiota in maintaining cognitive health [12]. Lower diversity may reflect reduced resilience of the gut ecosystem, making it more susceptible to external insults, inflammation, and metabolic dysfunction-factors that have all been implicated in neurodegeneration. Additionally, the reduced abundance of short-chain fatty acid (SCFA) producers, like Roseburia and Eubacterium, aligns with prior findings that link SCFAs to blood-brain barrier integrity and anti-inflammatory pathways [13]. Interestingly, despite pharmacological interventions with anti-dementia medications, microbial diversity remained unaltered, hinting at the possibility that current treatments do not address or reverse gut dysbiosis [14]. Moreover, constipation a symptom commonly observed in AD and linked to gut dysfunction was associated with lower microbial diversity, strengthening the gut-brain connection in this disease context [15]. Though causality cannot be inferred due to the cross-sectional design, the distinct microbial signatures across AD stages suggest the utility of the gut microbiome as both a biomarker and a modifiable therapeutic target [16]. Longitudinal studies are warranted to clarify whether dysbiosis precedes cognitive decline or evolves in parallel, and whether interventions such as dietary modulation, prebiotics, probiotics, or fecal microbiota transplantation may have a role in altering AD progression.

## Conclusion:

A clear association between reduced gut microbiome diversity and the progression of Alzheimer's disease is seen. Dysbiosis characterized by depletion of beneficial microbes and enrichment of pro-inflammatory taxa appears to worsen with cognitive decline. Thus, we show the gut microbiome as a potential biomarker and therapeutic target in the management of Alzheimer's disease.
